# Lipidated peptides and the limits of chemical control

**DOI:** 10.3389/fchem.2026.1807712

**Published:** 2026-05-04

**Authors:** Cesar Augusto Roque-Borda, Anamika Sharma, Fernando Rogério Pavan, Beatriz G. de la Torre, Fernando Albericio

**Affiliations:** 1 Vicerrectorado de Investigación, Universidad Católica de Santa María de Arequipa, Arequipa, Peru; 2 Peptide Science Laboratory, School of Chemistry and Physics, University of KwaZulu-Natal, Durban, South Africa; 3 Tuberculosis Research Laboratory, School of Pharmaceutical Sciences, São Paulo State University (UNESP), Araraquara, Brazil; 4 School of Laboratory Medicine and Medical Sciences, College of Health Sciences, University of KwaZulu-Natal, Durban, South Africa; 5 Department of Inorganic and Organic Chemistry, University of Barcelona, Barcelona, Spain

**Keywords:** albumin, lipidation, peptide, potency, selectivity

## Abstract

Peptide lipidation is widely employed to enhance the apparent biological performance of peptide-based systems by improving stability, membrane association, and systemic persistence. However, increased potency is often interpreted uncritically as evidence of improved molecular design. This Perspective highlights that lipidation can reshape peptide behaviour by partially shifting functional control from sequence-encoded molecular recognition toward context-dependent effects in which membrane interactions, carrier binding, supramolecular assembly, and residence time play an increasingly important role. Under such design-dependent conditions, enhanced activity may primarily reflect delivery- and exposure-driven amplification rather than improved intrinsic efficacy. While MIC values and endpoint assays remain valuable benchmarks of activity, they are insufficient on their own to guide rational optimization of lipidated peptides, as they conflate intrinsic activity with lipid-driven distribution and time-dependent effects. Accordingly, this Perspective argues not against lipidation itself, but for reframing lipidation as a deliberately controllable interaction element rather than a generic potency modifier, thereby restoring mechanistic interpretability and design robustness in peptide chemical biology.

## 1 Introduction

**FIGURE 1:**
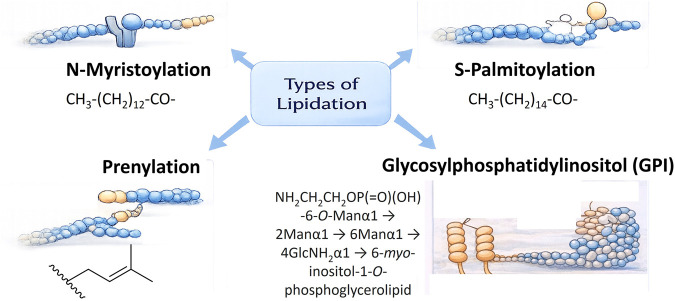
Lipidation as post-translational modifications (PTM) in proteins and peptides.

Peptides occupy a central position in chemical biology due to their structural modularity, synthetic accessibility, and capacity for highly specific biological interactions (Wang et al., 2022). Their intermediate size enables selective recognition processes that are often inaccessible to small molecules while avoiding the architectural complexity of full-length proteins. Despite these advantages, many peptide-based systems suffer from intrinsic limitations, including poor metabolic stability, rapid clearance, and limited membrane permeability, which constrain their functional and translational performance (Furman et al., 2015; Roque-Borda et al., 2026). To address these challenges, a range of chemical strategies has been developed like PEGylation, glycosylation, lipidation, mirror-image proteins, etc (Qi et al., 2024). Among all lipidation has emerged as one of the most widely applied and clinically validated approaches. Lipidation is a ubiquitous post-translational modification (PTM) characterized by the covalent attachment of hydrophobic lipid moieties to proteins and peptides (Figure 1), thereby regulating their membrane association, subcellular localization, stability, and signalling behaviour (Hang and Linder, 2011; Zhang and Bulaj, 2012). Prominent forms of lipid PTMs include N-myristoylation, S-palmitoylation, glycosylphosphatidylinositol (GPI) anchoring, and prenylation, each contributing in distinct ways to the spatial and functional organization of biomolecules within cellular environments (Zhang and Bulaj, 2012).

Table 1 highlights the representative examples from the literature highlight the impact of these modifications.

**TABLE 1:** Representative lipidated FDA approved peptides with engineered and predictable properties.

System	Design strategy	Key effect of lipidation	Core insight
Liraglutide/Semaglutide/Tirzepatide	Precisely tuned fatty acid length and linker geometry	Prolonged residence time via reversible albumin binding	Potency reflects controlled exposure and distribution, not enhanced receptor affinity
Insulin detemir	Single-site fatty acylation (myristic acid) enabling carrier association	Delayed absorption and reduced clearance	Robust clinical behaviour achieved through PK engineering rather than altered molecular recognition
Engineered lipidated AMPs (literature examples)	Balanced lipid length and charge to constrain aggregation	Increased membrane proximity with preserved selectivity	Aggregation can be bounded and programmable, yielding predictable ensemble behaviour

Lipidation typically involves the covalent attachment of hydrophobic moieties, most commonly long-chain fatty acids, but also sterols or lipid mimetics via defined conjugation strategies such as *N*-terminal modification, lysine side-chain functionalization, or linker-mediated attachment. Through these modifications the physicochemical properties of the peptides are fundamentally reshaped which frequently leads to prolonged systemic persistence, enhanced membrane association, and increased apparent biological potency (Menacho-Melgar et al., 2019; Xiao et al., 2025).

Clinically successful systems such as glucagon-like peptide-1 (GLP-1) analogues and insulin detemir exploit lipidation to achieve prolonged circulation through reversible albumin binding, while lipidated antimicrobial peptides (AMPs) utilize hydrophobic interactions to enhance membrane association and local concentration at biological interfaces. These examples underscore that lipidation is not a single mechanistic intervention, but a versatile design strategy capable of modulating multiple aspects of peptide behaviour (Knudsen et al., 2000; Kurtzhals et al., 2000; Lau et al., 2015; Makovitzki et al., 2008). Consequently, lipidation is frequently applied to improve the biological performance of peptides in therapeutic and chemical biology settings. At the physicochemical level, lipidation introduces a dominant hydrophobic domain that alters peptide solubility, conformational dynamics, and intermolecular interactions. These changes promote reversible binding to serum proteins, partitioning into lipid membranes, and in some cases supramolecular assembly, thereby reshaping peptide distribution and persistence in biological environments.

Beyond improving pharmacokinetic behaviour, lipidation can profoundly alter how peptides interact with biological environments. The introduction of a dominant hydrophobic domain shifts peptide behaviour away from purely sequence-encoded molecular recognition toward regimes in which interfacial interactions, supramolecular assembly, and carrier-mediated processes play an increasingly important role. In this context, activity may arise not from a single, well-defined molecular species, but from the collective behaviour of multiple interconverting states whose distribution depends on environmental variables such as membrane composition, protein binding, and formulation conditions (Hutchinson et al., 2017; Menacho-Melgar et al., 2019; Mu et al., 2025; Stachurski et al., 2022).

While lipidation reliably enhances apparent potency, this transformation introduces a design-dependent conceptual challenge. In some systems, increased activity reflects improved exposure, proximity effects, or extended residence time rather than enhanced intrinsic efficacy or molecular control. Thus, apparent potency must be understood as an emergent property that integrates both molecular recognition and system-level behaviour.

When lipid-mediated interactions are insufficiently constrained functional gains are accompanied by reduced predictability, mechanistic clarity, and robustness across biological contexts. Recognizing and managing this trade-off rather than avoiding lipidation altogether is essential for the rational and robust design lipidated peptide systems. While lipidation is widely used to enhance the apparent potency and durability of peptide systems, its effects are often interpreted through endpoint metrics that mask the underlying mechanisms of action. Importantly, these physicochemical transformations have direct implications for how potency should be interpreted. While lipidation reliably enhances apparent biological activity, a substantial fraction of this enhancement arises from changes in pharmacokinetic and Absorption, Distribution, Metabolism, and Excretion (ADME) related properties including increased systemic exposure, prolonged residence time, and altered biodistribution rather than from improvements in intrinsic target-binding affinity. This distinction is critical but often underappreciated and forms a central theme of this Perspective. In the following sections, we systematically examine how lipidation reshapes peptide behaviour across physicochemical, kinetic, and biological dimensions, and how these changes translate into apparent gains in potency. We further highlight the inherent trade-offs between enhanced exposure and loss of chemical and biological control, and outline design principles aimed at restoring predictability and robustness in lipidated peptide systems.

## 2 Potency gains and chemical control in lipidated peptides

Lipidation has emerged as one of the most effective strategies to enhance the apparent potency of peptides in chemical biology and therapeutic research (Hang and Linder, 2011; Kowalczyk et al., 2017; Pollaro and Heinis, 2010). By appending hydrophobic moieties viz., fatty acids, sterols, aromatic lipids, or lipid mimetics, poorly permeable peptides are transformed into molecules that persist longer in biological systems, enter cells more efficiently, and display dramatically improved functional activity (Hang and Linder, 2011; Pollaro and Heinis, 2010). These successes have made lipidation a near-default modification for peptides that underperform in biological assays (Ward et al., 2013). At the outset, it is important to distinguish between intrinsic potency, arising from direct molecular recognition and target-binding affinity, and apparent potency, which reflects the combined influence of binding, exposure, residence time, and distribution in a biological system. Lipidation primarily enhances the latter, and this distinction provides a conceptual framework for understanding both the benefits and limitations of lipidated peptides. Lipidation is a ubiquitous PTM that covalently appends hydrophobic lipid moieties to proteins and peptides, thereby modulating their membrane affinity, subcellular localization, stability, and signalling lifetime (Hang and Linder, 2011; Zhang and Bulaj, 2012). Common types of lipids PTMs are *N*-myristoylation, *S*-palmitoylation, glycosylphosphatidylinositol (GPI) and prenylation anchoring (Zhang and Bulaj, 2012). They act as dynamic regulators that tune protein-membrane interactions rather than simply serving as structural appendages.

On the other hand, synthetic lipidation is a pharmacokinetic engineering strategy wherein the lipid moiety is attached to the peptide to lower the renal clearance, enable albumin binding, and increase the effective concentration at the target site. Through these mechanisms, lipidation enhances systemic exposure and prolongs the duration over which peptides can engage their targets, often leading to substantial increases in observed biological activity without necessarily improving intrinsic binding affinity. Increasing local concentration at lipid interfaces and restricting diffusional freedom, lipidation often amplifies biological potency and signalling efficiency (Menacho-Melgar et al., 2019). These effects are fundamentally linked to system-level behaviour such as membrane partitioning, carrier-mediated transport, and reduced clearance rather than purely to improvements in molecular recognition. However, this gain in potency often comes at a cost in terms of chemical control, defined here as the degree to which biological activity can be predicted and rationalized from intrinsic molecular features such as peptide sequence, structure, and binding affinity under defined conditions. In this context, we distinguish between chemical control (predictability based on intrinsic molecular properties) and biological control (predictability of function within complex, variable biological environments). Lipidation challenges both forms of control by introducing additional layers of context dependence. Lipidation can shift peptide behaviour away from regimes dominated by intrinsic molecular recognition toward context-dependent behaviour, in which activity is increasingly influenced by external factors including membrane interactions, carrier protein binding, aggregation state, formulation, and exposure time (Jiang et al., 2018). As a result, the lipidated peptide no longer behaves as a single, well-defined chemical entity acting through a single mechanism. Instead, it becomes a context-responsive ensemble whose activity is co-determined by biological surroundings (Přáda Brichtová et al., 2025).

**FIGURE 2:**
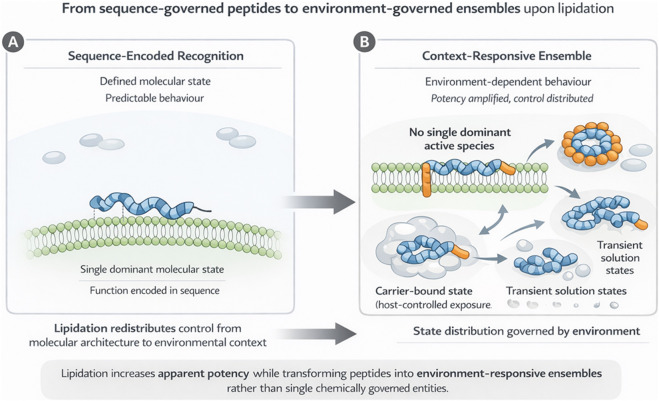
Schematic representation of the conceptual shift induced by lipidation in peptide systems. **(A)** Non-lipidated peptides function through sequence-encoded, chemically defined interactions, giving rise to a dominant molecular state. **(B)** Lipidation reshapes the physicochemical landscape, generating context-responsive ensembles in which activity emerges from membrane association, carrier binding, and proximity-driven effects governed by the environment.

This transition from a single defined molecular species to an ensemble of interconverting states represents a fundamental mechanistic shift. The distribution of these states and consequently the observed activity depends on environmental parameters such as protein binding, lipid composition, and concentration gradients, rather than solely on intrinsic binding interactions. This conceptual shift from sequence-governed molecular recognition to environment-responsive ensemble behaviour is schematically illustrated in Figure 2.

In classical chemical biology, control implies predictability which is a defined structure that produces a reproducible function under comparable conditions. Lipidation challenges this paradigm. Lipidation increases potency due to enhanced membrane interactions, albumin binding, or multimeric assembly formation in the form of stable oligomers (Mu et al., 2025). However, each mechanism varies depending upon administration route, specific lipidation strategy, and therapeutic target.

Importantly, these mechanisms are not universally additive or predictable; rather, they are highly context-dependent and may vary across *in vitro* and *in vivo* settings. For example, albumin binding may dominate systemic exposure in circulation, whereas membrane partitioning may govern activity at cellular interfaces. Such variability further complicates the interpretation of potency as a direct measure of molecular efficacy. Thus, lipidation does not simply “improve” peptides, it changes the rules by which they operate. Accordingly, potency gains in lipidated systems should be interpreted as emergent properties arising from the interplay of molecular recognition and pharmacokinetic context, rather than as direct indicators of improved intrinsic function.

## 3 Physicochemical consequences of peptide lipidation

Lipidation transforms peptides from sequence-encoded, hydrogen-bond-dominated molecules into amphiphilic systems whose behaviour is governed by both covalent and non-covalent interactions. Long-chain fatty acids are introduced into peptides through covalent attachment to alter their biological behaviour. Lipidation of peptides may be broadly categorized according to the type of covalent bond formed between the peptide backbone or side chain and the fatty acid, namely, amidation, esterification (S- or O-linked), and sulphur-based linkages such as ether or disulfide bonds. Of these, amidation and disulfide linkages are the most stable and widely used, while ester linkages are generally less robust (Myšková et al., 2023). While the *N*-terminal acylation remains the most commonly employed lipidation strategy, modification of lysine side chains by fatty acylation is also extensively investigated (Makowska et al., 2024). Peptide lipidation can prolong plasma circulation through albumin binding, a strategy exemplified in long-acting incretin-based therapeutics such as lipidated GLP-1 receptor agonists, where fatty-acid conjugation enables extended half-life without altering the peptide’s primary pharmacology (Mu et al., 2025; Zheng et al., 2024). The amphipathic character arises from the presence of a hydrophobic lipid domain which is independent of the secondary structure of the peptide alone (Straus and Hancock, 2006).

In non-lipidated peptides, intermolecular interactions are primarily governed by hydrogen bonding and electrostatics, favouring well-defined solution states and specific molecular recognition. However, in case of lipidated peptides, hydrophobic interactions and van der Waals forces are dominant (Almeida, 2014; Sharma et al., 2022). This shift redirects peptide behaviour toward interfaces and assemblies, favouring proximity-driven effects over high-affinity, lock-and-key recognition. The appended lipid chain introduces a strong propensity for hydrophobic clustering. Consequently, lipidated peptides readily self-associate into micelles, oligomers, or higher-order aggregates. These assemblies are not artefacts of formulation but represent thermodynamically accessible low-energy states stabilized by lipid-lipid and lipid-peptide interactions (Makowska et al., 2024; Menacho-Melgar et al., 2019). Lipidation also constrains conformational freedom by stabilizing membrane-compatible and aggregated states. Lipidation substantially alters the conformation of cationic peptides in terms of secondary structure thereby increasing their bio efficacy, whereas peptides bearing an overall negative charge respond differently to lipid chain incorporation (Straus and Hancock, 2006). In general, lipidation rather than enforcing a single bioactive structure, it redistributes the peptide’s conformational ensemble. The resulting energy landscape is characterized by multiple metastable states that are kinetically persistent and functionally competent. As depicted in Figure 2, lipidation expands the accessible conformational and supramolecular landscape, shifting function from a single dominant state to an ensemble of environment-dependent states. Lipid chains of suitable length introduce new binding equilibria that retard renal clearance and enhance systemic persistence, as described in the following section.

## 4 Mechanisms underlying potency enhancement

The covalent attachment of fatty acid moieties increases peptide lipophilicity, which in turn influences secondary structure, membrane affinity, and receptor interactions (Mu et al., 2025). However, these effects do not act independently rather, they collectively reshape peptide behaviour across multiple spatial and temporal scales, leading to enhanced apparent potency through a combination of physicochemical and pharmacokinetic mechanisms. To clarify these contributions, the mechanisms underlying potency enhancement can be broadly categorized into five interconnected domains (Mu et al., 2025): (i) membrane-mediated concentration effects, (ii) pharmacokinetic and exposure-driven effects, (iii) conformational and ensemble effects, (iv) Supramolecular assembly and higher-order organization, and (v) Integrating mechanisms: potency as an emergent property.

### 4.1 Membrane-mediated concentration effects

The introduction of a lipid chain constitutes major physicochemical perturbation that can reshape peptide behaviour across multiple length and time scales. The lipid tail acts as a membrane-anchoring element by inserting into lipid bilayers. This membrane tethering markedly increases the effective local concentration of the peptide at the cell surface, thereby amplifying biological responses through proximity-driven effects rather than through improvements in intrinsic molecular recognition or binding specificity. In this context, potency enhancement arises from increased effective concentration at the site of action, rather than from stronger ligand-receptor interactions. Importantly, this proximity-driven amplification can be either a deliberate design feature or an unintended consequence, depending on how lipidation is implemented. Once anchored onto the cell membrane, the lipidated peptide can either undergo passive insertion, endocytic pathways, or carrier-mediated transport. By concentrating the peptide at the membrane interface, lipidation can prolong receptor engagement and enhance signalling efficiency, even in cases where receptor binding affinity remains largely unchanged (Makowska et al., 2024; Myšková et al., 2023). Thus, membrane partitioning effectively converts a three-dimensional diffusion problem into a quasi-two-dimensional search process, increasing the probability of productive target engagement. In well-engineered systems, these effects are intentionally harnessed to improve efficacy and durability. As a result, while many lipidated peptides exhibit clear and systematic structure activity relationships (SAR), there are cases where potency enhancements arise from pharmacokinetic or spatial effects that are not captured by classical structure-activity analyses focused solely on receptor binding.

### 4.2 Pharmacokinetic and exposure-driven effects

Lipidation also exerts a profound influence on peptide pharmacokinetics. The increased hydrophobicity introduced by fatty acid conjugation promotes strong, yet reversible, binding to serum proteins (albumin). This interaction serves as a circulating depot, shielding the peptide from rapid enzymatic degradation, altering tissue distribution and reducing renal filtration, thereby improving *in vivo* potency, a principle that has been extensively validated in clinically successful long-acting lipidated peptide therapeutics (Bellavita et al., 2023; Zheng et al., 2024). Crucially, these effects extend the duration over which the peptide is available to interact with its target, thereby increasing time-integrated target engagement (residence time) without necessarily altering intrinsic binding affinity.

Consequently, lipidated peptides display markedly prolonged systemic half-lives, often extending from hours to days. Such predictable half-life extension has been achieved through rational control of lipid length, attachment site, and linker chemistry, relying on three primary mechanisms: (i) increased hydrodynamic radius to reduce renal elimination, (ii) steric shielding from proteases, and (iii) receptor-mediated endosomal escape and recycling.

### 4.3 Conformational and ensemble effects

From a conformational perspective, lipidated peptides do not operate through a single, well-defined active structure. Instead, their bioactivity emerges from the population of multiple metastable conformational states that are dynamically sampled in solution and at membrane interfaces. Lipidation biases the conformational ensemble toward membrane-compatible states without fully restricting conformational flexibility. This ensemble-based behaviour introduces an additional layer of complexity, whereby activity is governed by the distribution of accessible states rather than a single dominant conformation.

### 4.4 Supramolecular assembly and higher-order organization

At higher levels of organization, lipidation frequently promotes self-association phenomena, including micelle formation and oligomerization. When properly constrained, such supramolecular assemblies can be leveraged to enhance stability, exposure, and functional robustness. These assemblies can act as reservoirs or slow-release systems, further extending effective exposure and contributing to sustained pharmacological responses. However, uncontrolled aggregation may lead to nonlinear dose–response behaviour and reduced predictability (Mu et al., 2025; Přáda Brichtová et al., 2025).

### 4.5 Integrating mechanisms: potency as an emergent property

Taken together, these mechanisms highlight that potency enhancement in lipidated peptides is not driven by a single factor, but emerges from the interplay between molecular recognition, spatial localization, and pharmacokinetic behaviour. The central risk, therefore, is not potency amplification *per se*, but misattribution of its origin. A lipidated peptide may appear superior not because it binds better, but because it is delivered, retained, or protected more effectively. When these contributions are explicitly recognized and quantitatively understood, lipidation can be harnessed as a precise design tool; when they are overlooked, they can obscure SAR and compromise translational predictability.

This behaviour is exemplified by clinically approved GLP-1 analogues such as liraglutide and semaglutide, where fatty acid-mediated albumin binding extends systemic exposure and enhances apparent potency without substantially altering intrinsic receptor affinity.

## 5 Loss of chemical and biological control

The physicochemical mechanisms underlying lipidation explain why potency frequently increases, but they do not capture the trade-offs that may accompany this enhancement (Lin et al., 2023; Makowska et al., 2023). While above sections (Sections 2–4) establish how lipidation enhances apparent potency, the present section focuses on how these same mechanisms redistribute control away from intrinsic molecular features toward environmental and system-level variables. Beyond enhanced membrane association or prolonged systemic exposure, lipidation can alter the degree to which peptide behaviour remains chemically governed (Hang and Linder, 2011; Latendorf et al., 2019). In some lipidated systems, function becomes increasingly decoupled from sequence-encoded molecular recognition and progressively dominated by environmental variables. This redistribution of control across multiple physicochemical states and host-dependent interactions is summarized in Figure 2. When present, this transition is associated with reduced selectivity, mechanistic definition, and pharmacological predictability (Lai et al., 2022).

A primary manifestation of this loss of control arises from reduced selectivity thresholds. By introducing a dominant hydrophobic driving force, lipidation erodes electrostatic selectivity thresholds that many cationic peptides rely on to preferentially interact with anionic bacterial membranes while remaining largely excluded from zwitterionic host membranes (Beck et al., 2025; Lin et al., 2023; Paterson et al., 2017). Although lipidation can reduce membrane selectivity in some systems, multiple studies demonstrate that selectivity can be preserved through rational lipid design. Lipidation may reduce the free energy barrier for membrane insertion by introducing a dominant hydrophobic interactions that are less sensitive to surface charge. Mechanistically, lipidation lowers the energetic barrier for membrane insertion in a manner that is less dependent on electrostatic complementarity. As a result, membrane engagement can become less conditional on electrostatic complementarity and more permissive across lipid compositions. This shift does not necessarily result in immediate cytotoxicity under standardized assay conditions, but it compresses the energetic gap between productive and non-productive membrane interactions. In such cases, selectivity becomes highly dependent on local membrane composition, peptide concentration, and exposure time rather than intrinsic molecular discrimination (Lai et al., 2022; Rogers et al., 2021). Similar effects have been reported in lipidated antimicrobial peptides, where increasing lipid chain length enhances potency but reduces membrane selectivity, illustrating the trade-off between activity and control.

In parallel, lipidation can introduce dynamic aggregation equilibria that complicate the definition of a single active species, as lipidated peptides often populate ensembles comprising monomers, transient oligomers, micellar assemblies, and higher-order aggregates (Adak et al., 2024; Vicente-García and Colomer, 2021). The relative population of these states is governed by ionic strength, lipid availability, serum protein concentration, and formulation history, reflecting thermodynamically accessible assemblies rather than formulation artefacts. Under these conditions, biological activity therefore emerges from ensemble behaviour rather than a discrete molecular entity, rendering classical structure–activity relationships insufficient as measured potency reflects the context-dependent distribution of multiple species (Adak et al., 2024; Brown et al., 2018; Rounds and Straus, 2020).

This ensemble-driven behaviour can complicate mechanistic attribution, as activity in non-lipidated systems can often be linked to a defined interaction mode, such as pore formation, receptor binding, or intracellular target engagement. In lipidated peptides, similar phenotypic outcomes have been reported to arise arise from fundamentally different underlying states depending on environment (Juhl et al., 2021; Lin et al., 2023; Makowska et al., 2024). Thus, identical experimental readouts may correspond to distinct underlying mechanisms, reducing the transferability of mechanistic conclusions across systems (Makowska et al., 2024; Rounds and Straus, 2020).

Loss of control may be further influenced by strong interactions with host carrier proteins, as lipidated peptides frequently exhibit high affinity for serum albumin and lipoproteins that dominate their effective hydrodynamic radius, circulation time, and tissue distribution. While such binding can enhance systemic exposure, it also introduces host-dependent contributions to biodistribution. In this regime, the lipid moiety can become a dominant determinant of pharmacological behaviour, such that small changes in lipid structure produce disproportionate *in vivo* effects that are not captured by *in vitro* assays (Helmy et al., 2026; Menacho-Melgar et al., 2019; Mu et al., 2025).

At the pharmacological level, lipidation can decouple apparent activity from intrinsic efficacy, as enhanced potency may arise from increased residence time, altered tissue partitioning, or protection from clearance rather than from improved target engagement. This decoupling can complicate dose–response interpretation and increases sensitivity to formulation, administration route, and biological context. As reported in multiple studies, reproducibility across laboratories and experimental models may be is reduced, even when lipid-mediated effects dominate functional (Kopp et al., 2024; Menacho-Melgar et al., 2019; Wang et al., 2016).

Collectively, these observations suggest a shift in how function can be encoded in lipidated peptide systems: lipidation may transform peptides from chemically programmable entities into context-responsive systems whose behaviour is co-determined by environment, assembly state, and host interactions. Importantly, this loss of control is not an inherent flaw of lipidation, but a consequence of insufficiently constrained hydrophobic interactions. When explicitly managed, these same mechanisms can be harnessed to produce predictable and robust systems.

## 6 Potency as a misleading design criterion

The widespread adoption of lipidation as a peptide optimization strategy has been accompanied by a methodological simplification: increased potency is frequently interpreted as evidence of improved molecular design. However, as established in the preceding sections, potency in lipidated systems is an integrated outcome of molecular recognition, exposure, distribution, and time-dependent processes, rather than a direct measure of intrinsic efficacy.

In much of the current literature, in case of lipidated peptides reductions in MIC values, enhanced cellular activity, or prolonged *in vivo* exposure are collectively taken as indicators of successful structure-function refinement (Liu et al., 2025; Reinhardt and Neundorf, 2016; Van Gent et al., 2021). However, in lipidated systems, potency cannot be interpreted in isolation, as it conflates intrinsic molecular activity with delivery efficiency, residence time, and context-dependent amplification effects (Roque-Borda et al., 2026; Talha and Roque-Borda, 2026).

Treating potency as a primary design endpoint therefore obscures the mechanistic origin of activity and can lead to incorrect structure-function interpretations. In lipidated peptides, small changes in assay format such as inoculum density, ionic strength, serum supplementation, or pre-incubation time have been shown to produce substantial shifts in apparent potency without any alteration in peptide structure (Loffredo et al., 2021; Rounds and Straus, 2020; Van Gent et al., 2021). Under these conditions, potency remains a useful comparative metric, but increasingly reflects assay-specific amplification rather than intrinsic molecular performance.

The absence of kinetic information further limits the interpretability of potency metrics, which capture near-equilibrium outcomes while lipidated peptides often operate through time-dependent processes such as membrane accumulation, slow dissociation, and dynamic assembly (Fantner et al., 2010; Walker and Marty, 2022). A peptide that appears highly potent may therefore do so because it accumulates more efficiently or persists longer at the site of action, rather than because it binds more strongly. Without resolving association and dissociation kinetics, it is not possible to distinguish between improved intrinsic efficacy and exposure-driven effects. Consequently, optimization efforts guided primarily by potency risk selecting constructs that perform well under specific experimental timescales but fail under different biological conditions (Bertrand and Munoz-Garay, 2025; Knockenhauer and Copeland, 2024).

The conceptual trap is reinforced by reductionist structure-activity paradigms that assume incremental chemical modifications produce proportional and interpretable changes in activity (Ciulla and Gelain, 2023; Skowron et al., 2023). Lipidation challenges this assumption by introducing nonlinear and discontinuous effects, whereby variations in lipid chain length, attachment site, or linker chemistry can shift the dominant physicochemical regime, altering aggregation behaviour, membrane engagement, and protein binding (Lin et al., 2023; Rounds and Straus, 2020). When potency increases abruptly under such conditions, it is sometimes misattributed to optimized molecular recognition rather than to regime shifts in physical behaviour.

In such cases, abrupt increases in potency may reflect transitions between physical regimes (e.g., monomeric to aggregated states or solution-phase to membrane-associated behaviour) rather than true improvements in molecular recognition. Another contributing factor is the implicit normalization of context dependence, whereby variability in potency across laboratories or biological models is rationalized as biological complexity rather than recognized as a signal of limited control (Meurer et al., 2021; Parker et al., 2018).

In lipidated systems, such variability is not incidental but intrinsic to the design strategy, and therefore requires explicit characterization rather than implicit acceptance (Helmy et al., 2026; Makowska et al., 2024; Walker and Marty, 2022). This conflation of potency with design success has downstream translational consequences. Constructs selected for maximal activity may advance despite unresolved liabilities in selectivity, mechanism, or pharmacological behaviour. Accordingly, potency should be treated as an outcome requiring mechanistic validation, rather than as a standalone design principle. In lipidated peptide systems, increased activity should be interpreted as a signal for deeper interrogation, not as confirmation of successful optimization. When complemented by kinetic, mechanistic, and distribution-aware analyses, potency remains a valuable component of rational design.

## 7 Strategies to recover control in lipidated peptides

The loss of chemical and biological control observed in lipidated peptide systems does not arise from a single failure mode, but from the convergence of multiple physicochemical effects that operate across different length and time scales. Lipidation simultaneously alters membrane partitioning, assembly behaviour, carrier interactions, and pharmacokinetic profiles, coupling molecular function to environmental variables in a manner that cannot be resolved through sequence optimization alone (Kowalczyk et al., 2017; Menacho-Melgar et al., 2019; van Gent et al., 2021). As a consequence, empirical improvements in potency alone provide limited guidance for restoring predictability or mechanistic clarity.

**FIGURE 3:**
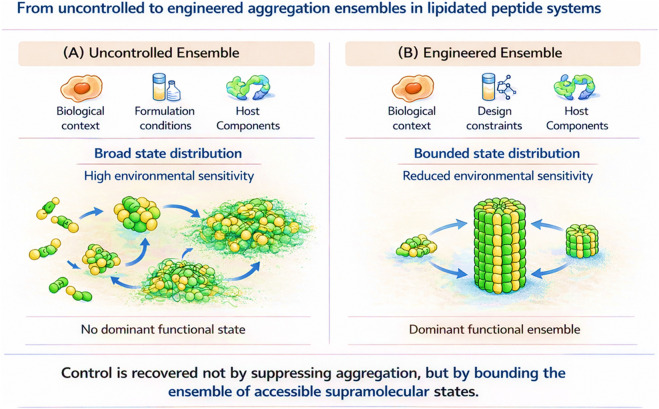
Uncontrolled versus engineered ensemble behaviour in lipidated peptides: **(A)** Uncontrolled lipidation produces broad, environment-dependent distributions of supramolecular states without a dominant functional species. **(B)** Engineered lipidation constrains aggregation into a bounded, robust ensemble, restoring functional predictability while preserving self-assembly.

Within this regime, control cannot be recovered by incremental tuning of hydrophobicity or charge, nor by conventional structure–activity relationships that assume a dominant molecular species. Instead, control must be imposed by design strategies that explicitly constrain how lipid-mediated interactions contribute to function. This requires shifting the design objective from maximizing activity Defining and regulating specific physicochemical contributions, including orientation, assembly, reversibility, and competition with host components. Accordingly, the following principles are reformulated as operational design criteria, each associated with experimentally accessible readouts rather than abstract heuristics. These considerations do not constitute new rules, but rather articulate recurring design logics that emerge when lipidation is treated as a programmable interaction element rather than as a potency-driven modification (Kowalczyk et al., 2017; Nielsen et al., 2020; Rounds and Straus, 2020). Restoring predictability and mechanistic coherence therefore requires treating lipidation as a regulatable design element, not as a generic potency modifier. Control can be reintroduced by constraining how lipid-mediated interactions contribute to membrane engagement, assembly, and biodistribution (Kowalczyk et al., 2017; Menacho-Melgar et al., 2019; Mu et al., 2025; Rounds and Straus, 2020; Van Gent et al., 2021):

- A first principle is decoupling lipid anchoring from peptide recognition: Spatial separation of lipid and functional domains using defined linkers allows membrane association to serve as a positioning mechanism rather than a determinant of activity. Operationally, linker length, flexibility, and polarity should be optimized against measurable parameters such as membrane residence time, receptor binding kinetics, and activity retention after lipid truncation. Designs that preserve activity upon partial lipid removal indicate successful decoupling.
- A second principle involves positional lipidation rather than terminal attachment: Internal or side-chain lipidation biases peptide orientation at membrane interfaces and limits uncontrolled insertion depth. This can be evaluated experimentally by comparing depth-of-insertion (e.g., fluorescence quenching, EPR, or MD simulations) and selectivity ratios across lipid compositions, rather than by potency alone. Control emerges when selectivity is maintained across membrane models despite increased hydrophobicity.
- A third principle is engineering aggregation as a defined variable: Rather than minimizing self-association, controlled designs restrict the ensemble of accessible supramolecular states through sequence-level modulation of hydrophobic patterning, charge distribution, and secondary structure propensity. Operationally, this requires correlating activity with defined aggregation windows (e.g., DLS, SAXS, cryo-EM) and rejecting constructs whose functional output varies sharply with concentration or formulation history. Functional robustness is indicated by stable activity across concentration and ionic-strength ranges. The contrast between uncontrolled aggregation and engineered, bounded ensembles is summarized in Figure 3.
- A fourth principle is reversibility as a temporal control parameter: Conditionally labile lipid attachments enable lipidation to function as a context-dependent switch. Designs should be benchmarked by comparing activity before and after lipid cleavage or exchange, enabling direct separation of lipid-driven amplification from intrinsic peptide function. Successful systems retain baseline activity while exhibiting localized, reversible enhancement.
- A fifth principle is integrating lipidation with complementary control strategies: In systems requiring high selectivity or precise molecular recognition, alternative or transient hydrophobic interactions may provide superior control. Determining when lipidation is suboptimal is itself a design outcome. Decision criteria should include whether lipidation improves robustness across environments or merely amplifies activity under narrow conditions. Identifying cases where lipidation is suboptimal is itself an actionable design outcome.

These principles recast lipidation as a controllable interaction element rather than a potency-driven modification. Functional robustness is recovered by constraining how hydrophobic interactions contribute to orientation, assembly, and biodistribution, not by eliminating them. In this framework, control is restored through measurable constraints on spatial, temporal, and supramolecular behaviour, enabling systematic design choices rather than empirical trial-and-error. As recently argued in the context of peptide chemistry, the uncritical accumulation of modified analogues frequently masks underlying regime shifts in molecular behaviour (Roque-Borda et al., 2025). Lipidation represents a particularly instructive case in which apparent gains in potency arise alongside a loss of chemical governance, necessitating design principles that reintroduce control at the level of interaction architecture rather than activity alone.

## 8 Conclusion

Lipidation remains a powerful and clinically validated strategy for enhancing the apparent potency and durability of peptide therapeutics. Importantly, numerous lipidated peptide drugs demonstrate predictable behaviour and coherent structure–activity relationships when lipid architecture and attachment geometry are deliberately controlled. The central implication of this review is therefore not that lipidation is intrinsically problematic, but that its success depends on explicitly managing how hydrophobic interactions govern partitioning, assembly, and biodistribution. When lipidation is treated as a programmable design variable rather than a generic potency modifier, peptide systems can be engineered for robustness, reproducibility, and mechanistic clarity rather than conditional activity.

## Data Availability

The original contributions presented in the study are included in the article/supplementary material, further inquiries can be directed to the corresponding author.
